# Selection of reference genes for quantitative real-time PCR expression studies of microdissected reproductive tissues in apomictic and sexual *Boechera*

**DOI:** 10.1186/1756-0500-4-303

**Published:** 2011-08-19

**Authors:** Marco Pellino, Timothy F Sharbel, Martin Mau, Samuel Amiteye, José María Corral

**Affiliations:** 1 Apomixis Research Group, Leibniz-Institute für Pflanzengenetik und Kulturpflanzenforschung (IPK), Corrensstraße 3, D-06466 Gatersleben, Germany

## Abstract

**Background:**

Apomixis, a natural form of asexual seed production in plants, is considered to have great biotechnological potential for agriculture. It has been hypothesised that de-regulation of the sexual developmental pathway could trigger apomictic reproduction. The genus *Boechera *represents an interesting model system for understanding apomixis, having both sexual and apomictic genotypes at the diploid level. Quantitative qRT-PCR is the most extensively used method for validating genome-wide gene expression analyses, but in order to obtain reliable results, suitable reference genes are necessary. In this work we have evaluated six potential reference genes isolated from a 454 (FLX) derived cDNA library of *Boechera*. RNA from live microdissected ovules and anthers at different developmental stages, as well as vegetative tissues of apomictic and sexual *Boechera*, were used to validate the candidates.

**Results:**

Based on homologies with *Arabidopsis*, six genes were selected from a 454 cDNA library of *Boechera*: RPS18 (Ribosomal sub protein 18), Efalpha1 (Elongation factor 1 alpha), ACT 2 (Actin2), UBQ (polyubiquitin), PEX4 (Peroxisomal ubiquitin conjugating enzyme) and At1g09770.1 (*Arabidopsis thaliana *cell division cycle 5). Total RNA was extracted from 17 different tissues, qRT-PCRs were performed, and raw Ct values were analyzed for primer efficiencies and gene ratios. The geNorm and normFinder applications were used for selecting the most stable genes among all tissues and specific tissue groups (ovule, anthers and vegetative tissues) in both apomictic and sexual plants separately. Our results show that *Boech*RPS18, *Boech*Efα1, *Boech*ACT2 and *Boech*UBQ were the most stable genes. Based on geNorm, the combinations of *Boech*RPS18 and *Boech*Efα1 or *Boech*UBQ and *Boech*Efα1 were the most stable in the *apomictic *plant, while *Boech*RPS18 and *Boech*ACT2 or *Boech*UBQ and *Boech*ACT2 performed best in the sexual plant. When subgroups of tissue samples were analyzed, different optimal combinations were identified in sexual ovules (*Boech*UBQ and *Boech*Efα1), in anthers from both reproductive systems (*Boech*ACT2 and *Boech*Efα1), in apomictic vegetative tissues (*Boech*Efα1 and *Boech*ACT2) and sexual vegetative tissues (*Boech*RPS18 and *Boech*Efα1). NormFinder ranked *Boech*ACT2 as the most stable in the apomictic plant, while *Boech*RPS18 was the best in the sexual plant. The subgroups analysis identified the best gene for both apomictic and sexual ovules (*Boech*RPS18), for anthers from both reproductive system (*Boech*Efα1) and for apomictic and vegetative tissues (*Boech*ACT2 and *Boech*RPS18 respectively)

**Conclusions:**

From a total of six tested genes, *Boech*RPS18, *Boech*Efα1, *Boech*ACT2 and *Boech*UBQ showed the best stability values. We furthermore provide detailed information for the accurate normalization of specific tissue gene expression analyses of apomictic and sexual *Boechera*.

## Background

Sexual reproduction in plants is a highly regulated process in which meiosis and syngamy initiate embryo and seed development. Aberrations in any step typically lead to abortion of seed development [1]. In contrast, apomixis (asexual reproduction through seeds [2]) is an alternative reproductive strategy in which aberrations to normal sexual processes are viable [3], and is found naturally in more than 400 species. Compared to sexual reproduction, apomixis is characterized by three developmental steps: the production of a meiotically unreduced egg cell (*apomeiosis*), *parthenogenetic *development of this egg cell without fertilization, and production of a functional endosperm with (*pseudogamy*) or without (autonomous) fertilization of the binucleate central cell of the ovule [4]. Importantly, apomictic seeds have embryos which are genetically identical to the mother plant. Hence, the successful introgression of apomixis into crop plants would greatly facilitate the fixation and propagation of genetic heterozygosity and associated hybrid vigour over successive generations, and could significantly reduce costs associated with hybrid seed production [5]. The biotechnological potential of apomixis has thus raised tremendous research interest.

Apomixis has repeatedly evolved from sex, and while the evolutionary origin and molecular nature of apomixis remain enigmatic, various hypotheses regarding specific genetic mechanisms have been proposed. One possible mechanism is de-regulation in the timing of sexual reproductive genes or pathways [4]. The switch from sexual to apomictic reproduction has also been associated with gene dosage effects during endosperm development [6]. Furthermore, the global regulatory effects of polyploidy and hybridity, both of which characterize virtually all asexual plants (and parthenogenetic animals), have been proposed as possible triggers for the switch from sex to apomixis [4,7]. More specifically, hybridity has been hypothesized to induce asynchronous expression of sexual reproduction genes to lead to apomixis [7].

Understanding patterns of differentially expressed genes is crucial for disentangling the complex regulatory networks which characterize sexual and apomictic seed production. Advances in cell isolation methods, in conjunction with next generation sequencing technology, have enabled global comparisons of gene expression patterns between sexual and apomictic reproductive tissues, and have provided support for deregulation of reproductive pathways in the switch from sex to apomixis [8,9]. The analysis of gene expression, however, requires sensitive, precise, and reproducible measurements for particular mRNA sequences in specific tissues. In this regard, quantitative real-time PCR (qRT-PCR) is at present the most extensively used method for validating genome-wide (e.g. microarray) expression data [10], due to its high sensitivity, specificity, and broad quantification range [11]. Although it is an extremely powerful technique, qRT-PCR requires strict normalization steps to compensate for several experimental variables that cannot be completely controlled (e.g. amount of starting material, enzymatic efficiency, differences in the transcription activity between cell or tissues) and which can influence reproducibility between experiments [12]. Accurate normalization of qRT-PCR results is thus essential for precise comparisons between samples. The standard approach for normalization of qRT-PCR data is the use of internal control or reference genes, often referred to as housekeeping genes (HKGs [13]). This class of genes encodes proteins that typically function in basic cell metabolism or maintenance, with constant expression levels and low levels of fluctuation between most tissues. Currently, the most common and well-described housekeeping genes used for the normalization of gene expression data include actin [14], glyceraldehyde-3-phosphate dehydrogenase (*G3PDH *[14]), ribosomal genes, cyclophilin, elongation factor 1-a (Efα1 [14-17] ), adenine phosphoribosyl transferase (*aprt *[18]) and tubulin [19]. Recently, Silveira *et al*. [20] established *Bbriz*UBCE, *Bbriz*E1F4A and *Bbriz*EF1 as the best reference genes for analyses of sexual and apomictic ovary tissues of the monocotyledon *Brachiaria*. Many studies have shown that standard housekeeping genes used as internal standards for the quantification of mRNA expression can indeed vary with the experimental conditions [15,21,22]. A well-tested housekeeping gene showing significant expression stability in a plant species or tissue type might not show the same stability if used in different experimental situations, species or tissues [13,15]. Reference genes therefore need to be properly validated for specific species, tissue types or reproductive modes when designing quantitative gene expression studies [23]. Furthermore, the use of a single housekeeping gene for qRT-PCR normalization is not recommended due to potential error, and it has been proposed that at least two or three housekeeping genes should be used in parallel as internal standards [12,21,24]. Thus it is essential that prior validation of all reference genes is performed to confirm their expression stability in particular experimental conditions or tissues/cells, in order to prevent inaccurate data interpretation and subsequent false conclusions.

The genus *Boechera *(Brassicacea) is becoming a model system for studying apomixis, being composed of both sexual and apomictic genotypes, the latter of which display quantitative variation for levels of apomictic seed production [3]. Importantly, the occurrence of diploid apomictic forms [25] in *Boechera *makes it possible to compare differences in gene expression between apomictic and sexual individuals without the concomitant effects of polyploidy. Moreover, as wild relatives of *Arabidopsis thaliana*, molecular genetic studies in *Boechera *are facilitated by the extensive genetic resources which have been developed for this model plant [26]. In addition, *Boechera *species have been used for comparative genomic analysis, including partial genome sequencing [27], genetic map construction [28] and transcriptome sequencing [8,9], and the entire genomes of *B. stricta *and *B. divaricarpa *are being sequenced (DOE Joint Genome Institute; http://www.jgi.doe.gov).

**Figure 1:**
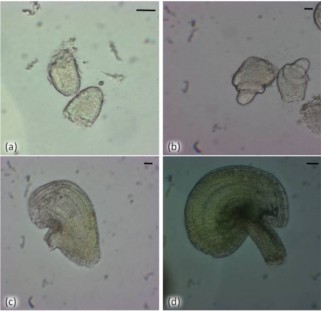
**Live microdissected *Boechera *ovules at multiple developmental stages**. (a) 1I to 1II; (b) 2II to 2IV; (c) 3II to 3III; (d) 3V to 4I [38]. Bar = 10 μm.

**Figure 2:**
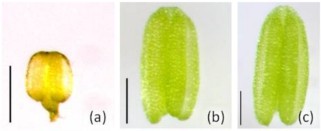
**Live microdissected *Boechera *anthers at multiple developmental stages**. (a) 7-8; (b) 9-10; (c) 11 [29]. Bar = 200 μm.

Considering the growing importance of this genus for evolutionary functional genomics, the aims of this work are to (1) validate the stability of some commonly used housekeeping genes, and (2) evaluate a new housekeeping gene for qRT-PCR analyses of microdissected reproductive tissues in apomictic and sexual members of the genus *Boechera*. We have identified one putative new HKG from our transcriptomic analyses of sexual and apomictic ovules at different developmental stages [8,9], and have additionally tested five known HKGs from *Arabidopsis *and other plant genera [Actin 2, s18 rRNA, elongation factor 1-α (Efα1), Pex 4 and Polyubiquitin 10; Table 1]. All HKGs were tested for stable gene expression patterns in both sexual and apomictic *Boechera *in various microdissected reproductive tissues including: four ovule stages (Figure 1; [9]), three anther stages (Figure 2; [29]) and four different tissues (flowers, leaves, roots, stems; Table 2).

**Table 1:** *Boechera*-specific qRT-PCT primers for tested HKGs

Gene identification/Gene description	Primer sequence 5'-3' forward/reverse	Amplicon size (bp)	Amplification efficiency ± SD *	EMBL Accession Number
BoechACT2/Actin 2	GTTCCACCACTGAGCACAATGTTACC/AGTCTTGTTCCAGCCCTCTTTTGTG	132	0.94 ± 0.003	FR846456
BoechEF1/Elongation factor-1 alpha	CCAAGGGTGAAAGCAAGGAGAGC/CACTGGTGGTTTTGAGGCTGGTATCT	75	0.96 ± 0.002	FR846458
BoechRPS18/Ribosomal protein S18	GCTGGGGAGTTATCTGCTGCTGAG/CTTGCCGTCTTTGTAATCCTTCTGC	117	0.94 ± 0.003	FR846460
BoechPEX4/Peroxin 4	TTTGCAGTTGACAGTTGGATCTTGTTC/TCGCTCGTGATGCCTATTCATCATAC	143	0.83 ± 0.009	FR846459
BoechAt1g09770.1/*Arabidopsis thaliana *cell division cycle 5	GCCATGATCTAAAAAGTTGGGACAAA/TATTCGTCACAACACATGCAAGGTTTA	145	0.88 ± 0.007	FR846457
BoechUBQ/Polyubiquitine	GGCTAAGATCCAGGACAAGGAAGGTAT/CTGGATGTTATAGTCAGCCAAAGTGCG	71	0.94 ± 0.004	FR851958

*Amplification efficiency was calculated using the miner algorithm [30] for both the means and corresponding SD: $\bar{x}=\sigma^{2}\cdot \overset{n}{\underset{i=\text{1}}{\sum }}\frac{\text{1}}{\sigma_{i}^{\text{2}}}x_{i}$[39]

**Table 2:** *Boechera *accessions, including ovule and anther stage-specific developmental characteristics

Species^a^	ID	Collection locality	Reproduction^b^	Ovule-anther stage^c^	Ovule development^d^	Anther stage^g^	Anther development^g^
*B. stricta*	MT49	Sagebrush Meadow, MT	Sex	a	Nucellus	7-8	Premeiotic PMC
				b	MMC^e ^formation	9-10	Meiotic PMC^f^
				c	Tetrad to degeneration	11	Microspore formation
				d	Fertilised ovules		
*B.divaricarpa*	MT15	Vipond Park, MT	Apomixis	a	Nucellus	7-8	Premeiotic PMC
				b	MMC^e ^formation	9-10	Meiotic PMC
				c	Tetrad to degeneration	11	Microspore formation
				d	Fertilized ovules		

^a ^Species identification were based upon silique orientation, trichome morphology, and cpDNA sequences [40]

^b ^Reproductive mode was confirmed using the flow cytometric seed screen [8,41]

^c ^See Figures 1 and 2 for images of each stage.

^d ^According to Schneitz et al [38]

^e ^MMC: Megaspore mother cell

^f ^PMC: Pollen mother cell stage

^g ^According to Armstrong *et al*.[29]

## Results

In order to identify optimal reference (HKG) genes in the genus *Boechera*, one candidate gene from a SuperSAGE dataset which was found to be uniformly expressed between apomictic and sexual *Boechera *accessions [8,9], and five previously-described HKGs (Actin 2, RPS18, Elongation factor 1-α, Pex 4 and UBQ) from *Arabidopsis *[14] and other plant species [15] were selected. Using the *Arabidopsis *genome database, in addition to 2 flower-specific *Boechera *cDNA libraries [8,9], we were able to design PCR primers in order to amplify, clone and sequence sections of the following six genes *Boech*ACT2, *Boech*PEX4, *Boech*PEX4, *Boech*RPS18, *Boech*Efα1, *Boech*At1g09770.1 and *Boechera *Polyubiquitin 10 (Table 1).

**Figure 3:**
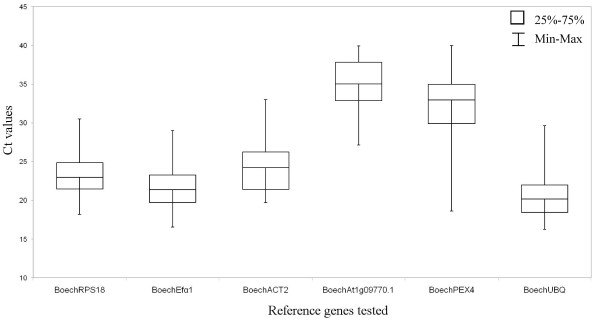
**Box-whisker plot**. Ct variation of each candidate reference gene among the different tissue samples.

The six candidate reference genes were evaluated for gene expression stability by qRT-PCR, using qRT-PCR primers designed in exonic regions from the cloned and sequenced *Boechera *homologues (Table 1). Based on cDNA analysis (not shown) and the dissociation curve (additional file 1) for each of the primer sets tested, a single PCR product with the expected size was amplified with HKG stability across both vegetative and microdissected reproductive tissues, and showed relatively tight Ct distributions for all 6 genes (Figure 3). Based upon the distribution of Ct values across different tissues, the HKGs could be split into low (*Boech*RPS18, *Boech*Efα1, *Boech*ACT2 and *Boech*UBQ) and high (*Boech*At1g09770.1 and *Boech*PEX4) values (Figure 3). Using the Miner algorithm [30], amplification efficiencies (E) were calculated to range between 0.74 ± 0.03% and 1.01 ± 0.1%. Expression ratios (R) were calculated, and amplification efficiencies and Ct values exported to the geNorm program as described by Vandesompele *et al*. [12] and normFinder program as described by Andersen *et al*. [31]. In order to evaluate gene stability, geNorm relies on the principle that two ideal control genes have the same expression ratio in all the samples despite cell type or experimental conditions. The program calculates two variables: the pairwise variation (V), which indicates the minimum number of reference genes required for a precise normalization, and the average pairwise variation of a particular gene compared to that of all other genes (M). Genes with the lowest M values have the most stable expression (Table 3). The normFinder program uses a model-based approach to select the genes with the minimum expression variation over the sample. Every gene is ranked with a stability value based on the intragroup variance and, if applicable, on the intergroup variance

**Table 3:** Summary of the 2 best HKG combinations for different tissues and reproductive system according to geNorm

Tissue	Recommended HKGs	M values	V2/3 values	Recommended HKGs (*Boech*RPS18 replaced by UBQ)	M values	V2/3 values
Apo all tissues	*Boech*RPS18 *Boech*Efα1	0.130	0.042	*Boech*UBQ *Boech*Efα1	0.120	0.120
Apo vegetative tissues	*Boech*Efα1 *Boech*ACT2	0.170	0.007	*Boech*UBQ *Boech*Efα1	0.220	0.120
Apo ovules	*Boech*RPS18 *Boech*Efα1	0.060	0.040	*Boech*UBQ *Boech*Efα1	0.055	0.131
Apo anthers	*Boech*ACT2 *Boech*Efα1	0.020	0.001	*Boech*ACT2 *Boech*Efα1	0.020	0.090
Sex all tissues	*Boech*RPS18 *Boech*ACT2	0.017	0.078	*Boech*UBQ *Boech*ACT2	0.200	0.100
Sex vegetative tissues	*Boech*RPS18 *Boech*Efα1	0.070	0.089	*Boech*UBQ *Boech*ACT2	0.220	0.100
Sex ovules	*Boech*RPS18 *Boech*ACT2	0.010	0.088	*Boech*UBQ *Boech*Efα1	0.010	0.057
Sex anthers	*Boech*Efα1 *Boech*ACT2	0.001	0.025	*Boech*Efα1 *Boech*ACT2	0.001	0.016

**Figure 4:**
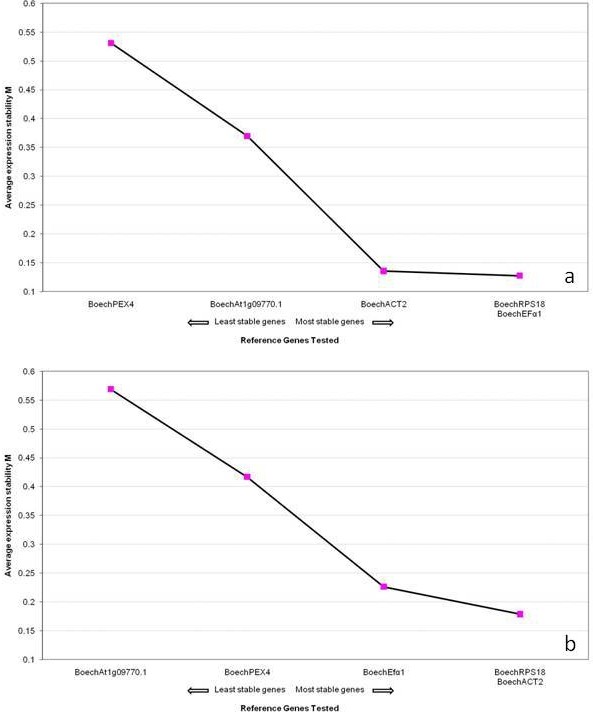
**Average expression stability values (M)**. Average expression stability values (M) of the control reference genes from geNorm, plotted from the least (left) to most stable (right) using UBQ as reference, from (a) apomictic vegetative and reproductive *Boechera *tissues, and (b) sexual vegetative and reproductive *Boechera *tissues.

**Figure 5:**
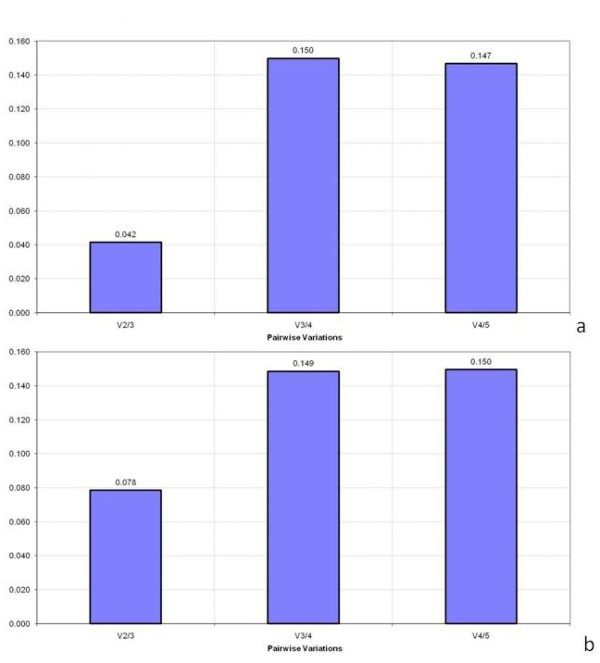
**Pairwise variation (V)**. Pairwise variation (V) of the selected reference genes in (a) apomictic and (b) sexual *Boechera*, as calculated from geNorm, from the most to least stable M values: (a) V2/3 - pairwise variation between the 2 most stable genes (RPS18 and Efα1) + the third most stable gene (Act2), V3/4 - addition of the fourth most stable gene (at1g09770.1), V4/5 - addition of the fifth most stable gene (Pex4); (b) V2/3 - RPS18, Act2 plus Efα1, V3/4 - plus Pex4, V4/5 - plus at1g09770.1.

The results based on geNorm show that three genes, *Boech*RPS18, *Boech*ACT2 and *Boech*Efα1, are the most stable in all tissues of both the apomictic and sexual accessions (Figure 4). The pairwise variation (V) values showed that for accurate normalisation, the two most suitable stable genes to employ are *Boech*RPS18 and *Boech*Efα1 for the apomictic accession, and *Boech*RPS18 and *Boech*Act2 for the sexual accession. With the addition of one more gene, pairwise variation (V2/3) values of 0.042 for the apomictic and 0.078 for the sexual accessions were obtained (Figure 5), values far below the cut-off of 0.15 suggested by Vandesompele *et al*. [12].

**Figure 6:**
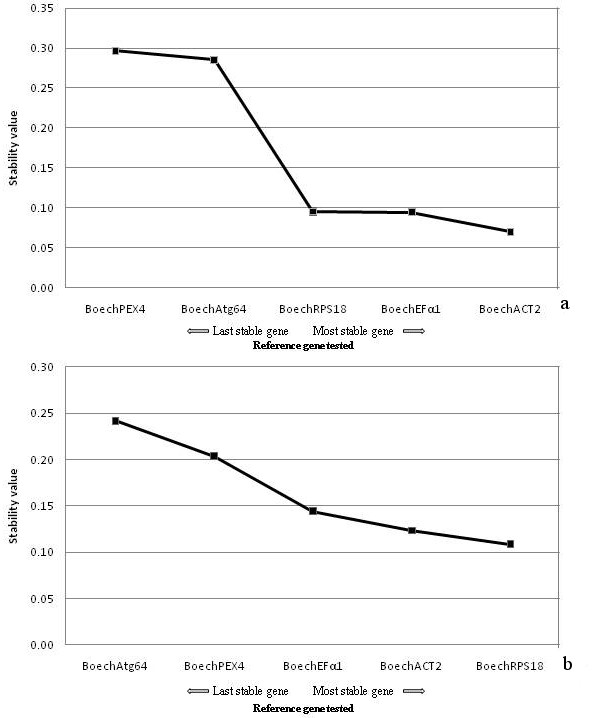
**NormFinder stability values**. Stability values of the control reference genes from normFinder, plotted from the least (left) to most stable (right) using UBQ as reference, from (a) apomictic vegetative and reproductive *Boechera *tissues, and (b) sexual vegetative and reproductive *Boechera *tissues.

NormFinder ranked, for the apomictic accession, *Boech*ACT2 and *Boech*Efα1 as the best genes with stability values of 0.070 and 0.094 respectively, and *Boech*RPS18 as the third best gene with a stability value of 0.095. For the sexual accession *Boech*RPS18 and *Boech*Act2 with stability values of 0.109 and 0.123 respectively were ranked as the most stable genes (Figure 6) in accordance with the result from geNorm.

In order to identify the best reference gene suitable for specific tissues, separate analyses were performed independently for vegetative tissues, and microdissected live ovules (all stages) and anthers (all stages; Table 2). As expected, *Boech*RPS18, *Boech*ACT2 and *Boech*Efα1 again exhibited high stability, but interestingly, based on geNorm, the stability values varied between tissue groups (Table 3). To validate this result, the analyses were repeated, but this time the two least stable genes (*Boech*PEX4 and *Boech*At1g09770.1) were removed from the set. In doing so, similar stability values (i.e. no effect on M) for *Boech*RPS18, *Boech*ACT2 and *Boech*Efα1 to those observed in the earlier analyses were obtained. In the subsequent analysis, normFinder confirmed the three candidates as the most stable ones. In 5 specific tissues the most stable ranked gene had also exhibited high stability using geNorm, and in only a single case one of the 2 best genes from geNorm were ranked as second and third by normFinder (Table 4). These data thus showed that *Boech*RPS18, *Boech*ACT2 and *Boech*Efα1 provide the best combination of HKGs for any tissue specific normalisation in *Boechera*.

**Table 4:** Summary of the 2 best HKG combinations for different tissues and reproductive system according to normFinder

Tissue	Recommended HKGs	Stability values	Recommended HKGs, (*Boech*RPS18 replaced by UBQ)	Stability values
Apo all tissues	*Boech*ACT2	0.07	*Boech*ACT2	0.085
	*Boech*Efα1	0.094	*Boech*Efα1	0.188
Apo vegetative tissues	*Boech*ACT2	0.073	*Boech*ACT2	0.22
	*Boech*Efα1	0.105	*Boech*Efα1	0.116
Apo ovules	*Boech*RPS18	0.015	*Boech*Efα1	0.014
	*Boech*Efα1	0.015	*Boech*UBQ	0.082
Apo anthers	*Boech*ACT2	0.007	*Boech*Efα1	0.106
	*Boech*Efα1	0.007	*Boech*ACT2	0.108
Sex all tissues	*Boech*RPS18	0.109	*Boech*ACT2	0.053
	*Boech*ACT2	0.123	*Boech*UBQ	0.065
Sex vegetative tissues	*Boech*RPS18	0.014	*Boech *ACT2	0.13
	*Boech*Efα1	0.015	*Boech*Efα1	0.134
Sex ovules	*Boech*RPS18	0.01	*Boech*Efα1	0.008
	*Boech*ACT2	0.151	*Boech*ACT2	0.039
Sex anthers	*Boech*Efα1	0.001	*Boech*Efα1	0.086
	*Boech*ACT2	0.001	*Boech*ACT2	0.088

Further analyses were done to identify the two best HKGs common to both apomictic and sexual reproductive modes. In removing Efα1 from the gene set of the apomictic accession, geNorm identified *Boech*RPS18 and *Boech*ACT2 as the most stable genes with M = 0.24 and V2/3 = 0.073, a result was also confirmed by NormFinder. In this case all samples were divided into two groups according to reproductive mode and analyzed simultaneously in order to find the best genes throughout all samples. *Boech*RPS18 and *Boech*ACT2 were the most stable genes with stability values of 0.054 and 0.078. *Boech*RPS18 and *Boech*ACT2 can therefore be used independently of reproductive mode, and should be chosen in cases where the reproductive mode of the plant under study is uncertain.

To ascertain the stability and suitability of Ubiquitin (*Boech*UBQ) in all tissues of *Boechera*, the R values of all test genes were recalculated with *Boech*RPS18 as control, and analysed. As expected geNorm showed *Boech*UBQ to be the most stable gene in both apomictic (M = 0.11) and sexual (M = 0.20) *Boechera*. Based upon calculations of V, the data reported here show that *Boech*UBQ and *Boech*Efα1 (V2/3 = 0.12) provide accurate normalisation for apomictic genotypes, whereas *Boech*UBQ and *Boech*Act2 (V2/3 = 0.10) are more appropriate for sexual *Boechera*. NormFinder instead ranked *Boech*UBQ as third best gene (stability value = 0.194) for the apomictic plant and the second best (stability value = 0.065) for the sexual.

## Discussion

Based upon the transcriptional profiles of the 6 housekeeping genes tested in this study, and geNorm and normFinder analyses of different vegetative and reproductive tissues of sexual and apomictic *Boechera*, we conclude that ribosomal subunit protein 18 (*Boech*RPS18), elongation factor-1 (*Boech*Efα1), Actin 2 (*Boech*ACT2) and Polyubiquitine (*Boech*UBQ) are the most stable. Although all 4 genes show significant stability, their values (M) varied depending on either specific tissue and/or reproductive system. Considering these criteria and pairwise variation (V), we propose optimal combinations of reference genes for normalization of gene expression data in transcriptome analyses of different tissues (Table 3). Using normFinder we were able to verify the stability of our candidates by using a different algorithm. Interestingly, in 11 analyses out of 16, the first HKG to be ranked as most stable by NormFinder was also found in the best combination provided by geNorm. In 5 cases the second best HKG to be ranked by normFinder was included in the best couple provided by geNorm, while the remaining one was ranked as the third best. These discrepancies could be explained by the fact that geNorm and normFinder use two different algorithms for the evaluation of the best HKGs. geNorm provides the two genes that have the most similar expression profiles through a stepwise elimination of the least stable genes in the sample, while normFinder instead uses a model-based approach to calculate a stability value which represents the expression variation of the gene throughout the sample. Differences in ranking when using these two programs have previously been found (see [32,33]). Considering the errors that could result from single HKG normalization strategy [12] and that at least one gene from the best couple identified by geNorm was always ranked in the first 2 positions by normFinder, we suggest using the specific pair of genes recommended by geNorm (Table 3). As validation for our choice of genes, the best couples of HKGs (according to geNorm) were chosen for normalizing the expression of 4 SuperSAGE tags that had previously showed reproduction specific expression between 2 ovule developmental stages in *Boechera*. qRT-PCR were performed and the relative expression of the 4 tags was normalized against *Boech*RPS18, *Boech*ACT2 and *Boech*Efα1 in the best combination according to geNorm, using the REST2009 software [34]. The result of the normalization was consistent with the SuperSAGE expression data (See additional file 2). We hypothesize that interactions between the 4 best housekeeping genes identified here are minimized since each is involved in independent cellular processes.

## Conclusions

This work is the first in depth analysis of reference genes in a dicot plant with both sexual and apomictic reproductive forms (*Boechera*) and, more importantly, the first report of a housekeeping gene analysis on live microdissected ovules and anthers. These data provide an important tool for transcriptomal analyses of reproductive tissues in *Boechera*, an excellent model system for the study of apomixis.

## Methods

Two *Boechera *accessions, a sexual diploid *B. stricta *and a facultative apomictic diploid *B. divaricarpa *were selected for the analyses (Table 2). Seedlings of these accessions were grown and maintained in a phytotron at the IPK under controlled environmental conditions (day: 16 h, 21°C; night: 8 h, 16°C; humidity 70%).

Six candidate housekeeping genes were selected, 5 previously-described HKGs in other plant genera and 1 new HKG which appeared to be stably expressed in a SuperSAGE dataset (Table 1; [8,9]). The gene to which the selected SuperSAGE tag sequence corresponded was found via a BLAST search [35] to two flower-specific (sexual and apomictic) *Boechera *cDNA libraries which were sequenced using 454 (FLX) technology [8]. The corresponding *Boechera *cDNA (Polyubiquitine, Table 1) was annotated using a homology search to the *Arabidopsis *genome (http://www.arabidopsis.org). *Arabidopsis *homologues to the 5 known HKG's were similarly identified, and these were BLASTed [35] to the *Boechera *cDNA libraries (E-value < 3e-024 and 2e-019 for the apomictic and sexual 454 cDNAs respectively) to obtain corresponding *Boechera*-specific gene sequences. PCR primers were then designed for DNA sequencing of the identified genes using DNASTAR Lasergene^® ^Primer Select (http://www.dnastar.com/products/lasergene.php).

DNA was extracted from 100 mg of leaf tissue from each plant using a Qiagen Dneasy^® ^Plant Mini Kit (QIAGEN, Hilden, DE) according to the manufacturer's instructions. For all HKGs, PCR reactions (10 μl) were mixed as follows: 25 ng of DNA, 1 μl of PCR Buffer II, 10 pmol for each primer, 0.025 U of AccuPrime™ Taq DNA Polymerase High Fidelity (Invitrogen, Carlsbad, CA) with 3.5 mM of MgCl_2 _and 4.95 μl of H_2_O. PCR reactions were performed in a Mastercycler ep384 (Eppendorf, Hamburg, DE) using the following touchdown thermal cycling profile: 94° for 10 min; 9 cycles of 94° for 15 sec, 65° for 15 sec (1 degree decrease in temperature every cycle with a final temperature of 54°), 72° for 30 sec; 35 cycles of 94° for 30 sec, 57° for 15 sec, 68° for 2 min 30 sec; and a final 68° for 15 min. Each PCR product was cloned into a TOPO TA Cloning^® ^(Invitrogen) vector according to the recommendation of the supplier. Eight clones per product were confirmed by DNA sequencing using Sanger Sequencing methods on an ABI 3730 xL platform (Applied Biosystem. Carlsbad, California) and analyzed using the DNASTAR Lasergene^® ^SeqBuilder and MegAlign programs.

qRT-PCR Primers were designed using DNASTAR Lasergene^® ^PrimerSelect, with all amplification products targeted between 70 and 160 bp, and melting temperatures between 58° to 63° C. The newly-designed primers were checked using the following PCR (20 μl) protocol: 25 μg of genomic DNA, 2 μl of 10 x reaction buffer, 20 pmol for each primer, 0.5 u/μl of BioTAQ DNA Polymerase (Bioline GmbH, Luckenwalde, DE), 2.5 nM of dNTPs, 2 mM of MgCl_2_, 11.1μl of water. We used the following thermal cycling profile: 94°C for 3 min, 35 cycles of 94°C for 30 sec, 59°C for 15 sec, 68°C for 1 min and finally 70°C for 7 min. The size of all PCR products was verified on a 1.5% agarose gel.

Total RNA was isolated from 4 different tissues (leaf, root, stem and flower) harvested from 2 biological replicates of both *Boechera *accessions (Table 2) using the Qiagen Rneasy^® ^Plant Mini Kit following the manufacturer's instructions. The isolated RNA was treated with Qiagen Rnase-Free DNase according to the producer's protocol in order to eliminate any contaminating traces of DNA. A second purification step was performed using a Qiagen^® ^Rneasy Mini Kit to eliminate contaminating polysaccharides, proteins and the DNase enzyme. The final concentration and quality was checked using an Agilent Technologies 2100 Bioanalyzer NanoChip (Agilent Technologies, - Santa Clara, CA, United States).

The gynoecia of sexual and apomictic *Boechera *were dissected from flowers at the megasporogenesis stage in a 0.55 M sterile mannitol solution between 7:30 am and 9:00 am each day. Microdissection was performed in a sterile laminar air flow cabinet under a stereoscopic microscope (Stemi 1000; Carl Zeiss). Ovules at 4 different developmental stages (Table 2 and Figure 1) and placental tissues were then collected under an inverted microscope (Axiovert 200 M; Carl Zeiss), in sterile conditions using sterile glass needles (self made using Narishige PC-10 puller). For each developmental stage approximately 20 ovules and 1 mm^2 ^of ovary tissue were collected in separate sterile Eppendorf tubes containing 200 μl of RNA stabilizing buffer, using a glass capillary (internal diameter 150 μm) interfaced to an Eppendorf Cell Tram Vario. Anthers at corresponding flower developmental stages 8-10 [29] (Figure 2) were selected for extraction of total RNA. Approximately 30 anther heads per sample were dissected from fresh whole flower buds and stored in RNA stabilizing buffer (RNA later; Sigma-Aldrich) under a stereoscopic microscope (Zeiss Stereo Discovery V12) using sterile glass needles. RNA was extracted using a Qiagen PicoPure Isolation Kit and purified of contaminating DNA using Qiagen RNase-Free DNase.

First strand cDNA was synthesised from 10 ng starting RNA with a RevertAid™H Minus First Strand cDNA Synthesis Kit (Fermentas) using an oligo(dT)18 primer following the manufacturer's instructions. The resultant concentration was checked using a PicoGreen^® ^dsDNA Quantitation Kit (Invitrogen) with a NanoDrop^® ^ND-3300 Spectrofluorometer (NanoDrop). qRT-PCR reactions were performed on an ABI-PRISM 7700 HT FAST Real-Time PCR System (Applied Biosystems) with the following cycling profile: 50°C for 2 min, 95°C for 10 min; 40 cycles of 95°C for 15 sec, 60°C for 1 minute. 10 μl reactions were performed using the following master mix: 5 μl of SYBR I Master Mix buffer, a total of 16.6 pmol for both sense and anti-sense primers, 2.5μl of water and 1.5 μl of cDNA. A melting curve gradient was obtained from the product at the end of the amplification for checking amplicon quality. cDNA samples derived from somatic tissues (leaf, root, stem and flower) were run in a serial dilution range of 5, 2.5, 1.25, 0.625 and 0.312 ng. All samples were run in triplicate with the control gene included in each plate. Due to low amounts of starting cDNA material from the micro-dissected ovules, a dilution range of 1, 0.5, 0.25, 0.125, 0.062 ng was used. Candidate and control genes were run simultaneously in two replicates with 4 ovule stages and 3 anther head stages for both sexual and apomictic accessions. *Boechera *Polyubiquitin 10 was selected as control gene due to its extensive use and proven reliability as a reference control in *Boechera *[8], *Arabidopsis *[14] and other plants [36]

Considering instrument background fluorescence, Crossing Point (Cp) is defined as the point at which sample fluorescence rises significantly above the background fluorescence characteristic of a particular detection system, and it is used as a measure for the starting copy numbers of the target gene. For every cDNA, the mean expression level and standard deviation for each set replicate was calculated. In cases where Cp values between replicates of the same gene diverged by more than one unit, as measured from cDNAs extracted from micro-dissected tissues, two additional replicates of that particular gene were performed under the same experimental conditions. The corresponding qPCR efficiencies were determined by the Miner algorithm [30]. To quantify gene expression in comparison to a reference gene, the relative expression ratio (R) was determined using the ΔΔCt method as described by Pfaffl [37]. The obtained R values for all the genes were transferred into the geNorm program (http://medgen.ugent.be/~jvdesomp/genorm/) for calculation of the expression stability as described by Vandesompele *et al*. [12]

For validation of the best HKGs, 4 SuperSAGE Tags that had shown reproductive mode-specific expression in cDNA between ovules at the second and fourth developmental stage were selected (see additional file 2). qRT-PCR reactions of cDNA from apomictic and sexual ovules at stages 2 and 4 were performed on an ABI-PRISM 7700 HT FAST Real-Time PCR System (Applied Biosystems) with the following cycling profile: 50°C for 2 min, 95°C for 10 min; 40 cycles of 95°C for 15 sec, 60°C for 1 minute. 10 μl reactions were performed using the following master mix: 5 μl of SYBR I Master Mix buffer, a total of 16.6 pmol for both sense and anti-sense primers, 2.5μl of water and 1.5 μl of cDNA diluted to 0.5 ng. Genes of interest and HKGs were run simultaneously in triplicate. For every cDNA, the mean expression level and standard deviation for each set replicate was calculated. The corresponding qPCR efficiencies were determined using the Miner algorithm [30]. The expression data were normalized according to the REST algorithm using the REST2009 software [34]

## Competing interests

The authors declare that they have no competing interests.

## Authors' contributions

MP was responsible for the experiment, microdissection, sample preparation, bioinformatics analysis of the sequences tested, qRT-PCR assay and drafting the manuscript. MM contributed with tissue isolation, RNA and cDNA sample preparation. SA, JMC and TFS participated as supervisors in the study design, analyses and writing. All authors read and approved the final manuscript.

## Supplementary Material

Additional file 1 **Dissociation curves**. Dissociation curves of the 9 amplicons after the qRT-PCR reactions, all showing one peak. Click here for file

Additional file 2 **SuperSAGE tags validation**. Validation of 4 specifically-expressed SuperSAGE tags from apomictic ovules. Click here for file
