# Supplementary figures and images for: Selection of reference genes for quantitative real-time PCR expression studies of microdissected reproductive tissues in apomictic and sexual Boechera
Source: BMC Res Notes. 2011 Aug 19;4:303. doi: 10.1186/1756-0500-4-303 (PMC3171723; doi:10.1186/1756-0500-4-303)

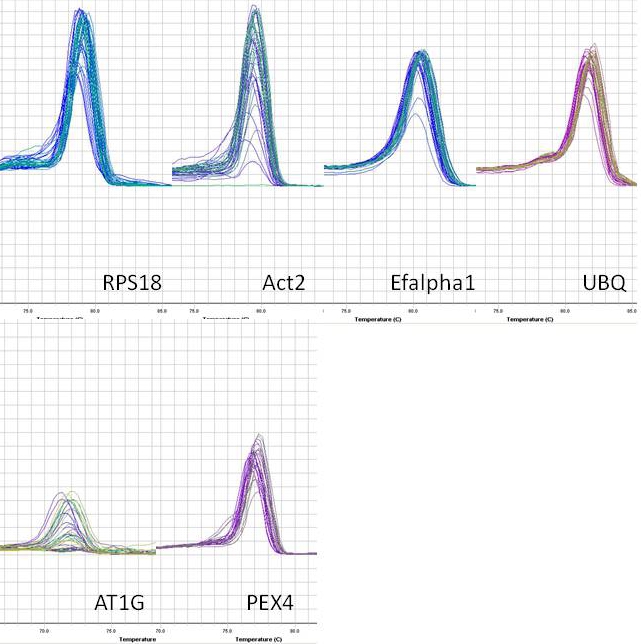

Supplement: Additional file 1 — Dissociation curves. Dissociation curves of the 9 amplicons after the qRT-PCR reactions, all showing one peak. [file 1756-0500-4-303-S1.JPEG]
