# Supplementary material for: Selection of reference genes for quantitative real-time PCR expression studies of microdissected reproductive tissues in apomictic and sexual Boechera
Source: BMC Res Notes. 2011 Aug 19;4:303. doi: 10.1186/1756-0500-4-303 (PMC3171723; doi:10.1186/1756-0500-4-303)
Supplement: Additional file 2 — SuperSAGE tags validation. Validation of 4 specifically-expressed SuperSAGE tags from apomictic ovules. [file 1756-0500-4-303-S2.PDF]

Validation of 4 specifically expressed SuperSAGE tags from apomictic ovules

Reproduction	Locus name	SuperSage tag	Relative SuperSAGE Expression [a]		HKGs Normalization [b]		
			Expression	P value	Expression	P value	Result
Apomictic	AT2G29140	CATGCCAATGTGTAGAATGCTTTTTA	8.500	0.0079	14.877	0.000	Up regulated in stage 2
	AT3G54670	GATCTATTCGAGAAATGCAGATGAAG	1.523	0.0179	9.481	0.000	Up regulated in stage 2
Sexual	AT1G15880	GATCAAAAACATAAATATTGTGTACA	0.235	0.012	0.369	0.000	Down regulated in stage 2
	AT1G51760	CATGTTGTTTTGCATTCCAAGTCTCT	0.714	0.003	0.514	0.000	Down regulated in stage 2

a Relative expression between ovule at stage 2 and 4

b According to REST software. Using the best HKG combination for apomictic ovule according to geNorm
